# Supplementary material for: Efficacy of Granulocyte Colony-Stimulating Factor in Acute on Chronic Liver Failure: A Systematic Review and Survival Meta-Analysis
Source: J Clin Med. 2023 Oct 16;12(20):6541. doi: 10.3390/jcm12206541 (PMC10607065; doi:10.3390/jcm12206541)
Supplement: Supplementary file 1 [file jcm-12-06541-s001.zip › jcm-2621201-supplementary.pdf]

MEDLINE database
Search: ACLF-Paper
Last Saved: 21.05.2023

("Liver Failure" [Mesh] OR "Failure, Acute on chronic Liver" OR "ACLF" OR "acute-on-chronic liver failure" OR "acute on chronic liver failure") AND
"Granulocyte Colony-Stimulating Factor" [Mesh] OR "G-CSF")

83 entries.

Cohrane DataBase
Search Name: ACLF-Paper
Last Saved: 21.05.2023
Comment:

ID	Search
#1	Title Abstract Keyword: Liver Failure
#2	Title Abstract Keyword: Failure, Acute on chronic liver
#3	Title Abstract Keyword: ACLF
#4	Title Abstract Keyword: acute on chronic liver failure
#5	Title Abstract Keyword: acute-on-chronic liver failure
#6	#1 OR #2 OR ## OR #4 OR #5
#7	Title Abstract Keyword: "granulocyte colony stimulating factor"
#8	Title Abstract Keyword: G-CSF
#9	#7 OR #8
#10	#6 AND #9

107 entries

SCOPUS Database
Search Name: ACLF paper
Last Saved: 21.05.2023

(TITLE-ABS-KEY("Liver Failure") OR TITLE-ABS-KEY("Failure, Acute on chronic Liver") OR TITLE-ABS-KEY("ACLF") OR TITLE-ABS-KEY("acute-on-chronic liver failure") OR TITLE-ABS-KEY("acute on chronic liver failure")) AND (TITLE-ABS-KEY("Granulocyte Colony-Stimulating Factor") OR TITLE-ABS-KEY("G-CSF"))

533 entries

Suppl.Figure 1. Overall survival, sensitivity analysis Risk of bias

Study or Subgroup	log[Hazard Ratio]	SE	Weight	Hazard Ratio IV, Random, 95% CI
**3.1.1 Overall Survival, main analysis**				
Duan XZ 2013	−0.6733	0.3384	27.9%	0.51 [0.26, 0.99]
Engelmann C 2021	0.0516	0.194	40.3%	1.05 [0.72, 1.54]
Garg V 2012	−0.821	0.4389	0.0%	0.44 [0.19, 1.04]
Saha BK 2017	−0.9163	0.7674	0.0%	0.40 [0.09, 1.80]
Tong J 2022	−0.6349	0.2904	31.8%	0.53 [0.30, 0.94]
**Subtotal (95% CI)**			**100.0%**	**0.69 [0.41, 1.16]**
Heterogeneity: Tau^2 = 0.14; Chi^2 = 5.74, df = 2 (P = 0.06); I^2 = 65%				
Test for overall effect: Z = 1.40 (P = 0.16)				
**Total (95% CI)**			**100.0%**	**0.69 [0.41, 1.16]**
Heterogeneity: Tau^2 = 0.14; Chi^2 = 5.74, df = 2 (P = 0.06); I^2 = 65%				
Test for overall effect: Z = 1.40 (P = 0.16)				
Test for subgroup differences: Not applicable				

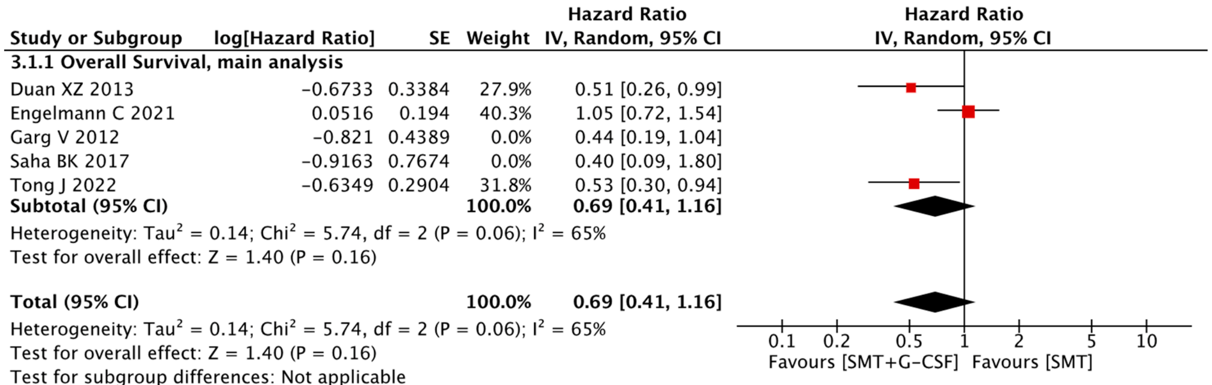

Duan XZ [29], Engelmann C [19], Garg V [30], Saha BK [31], Tong J [32]

Suppl.Figure 2. Overall survival, sensitivity analysis ACLF criteria

Study or Subgroup	log[Hazard Ratio]	SE	Weight	IV, Random, 95% CI
**3.1.1 Overall Survival, main analysis**				
Duan XZ 2013	−0.6733	0.34	31.6%	0.51 [0.26, 0.99]
Garg V 2012	−0.82	0.44	18.9%	0.44 [0.19, 1.04]
Saha BK 2017	−0.92	0.77	6.2%	0.40 [0.09, 1.80]
Tong J 2022	−0.6349	0.2904	43.3%	0.53 [0.30, 0.94]
**Subtotal (95% CI)**			**100.0%**	**0.50 [0.34, 0.72]**
Heterogeneity: Tau^2 = 0.00; Chi^2 = 0.21, df = 3 (P = 0.98); I^2 = 0%				
Test for overall effect: Z = 3.66 (P = 0.0003)				
**Total (95% CI)**			**100.0%**	**0.50 [0.34, 0.72]**
Heterogeneity: Tau^2 = 0.00; Chi^2 = 0.21, df = 3 (P = 0.98); I^2 = 0%				
Test for overall effect: Z = 3.66 (P = 0.0003)				
Test for subgroup differences: Not applicable				

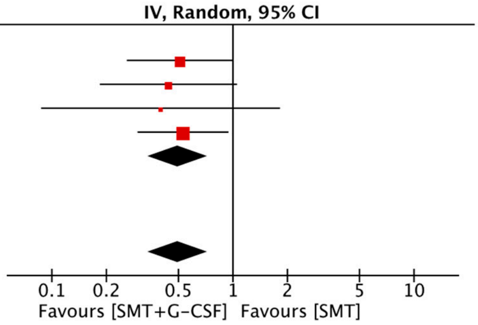

Duan XZ [29], Garg V [30], Saha BK [31], Tong J [32]

Suppl.Figure 3.

a) Overall survival, sensitivity analysis follow-up 30 days

Study or Subgroup	log[Hazard Ratio]	SE	Weight	Hazard Ratio IV, Random, 95% CI
Engelmann C 2021	0	0.2277	31.5%	1.00 [0.64, 1.56]
Garg V 2012	−0.8916	0.513	24.3%	0.41 [0.15, 1.12]
Saha BK 2017	−0.478	0.8823	15.6%	0.62 [0.11, 3.49]
Tong J 2022	−1.6094	0.3537	28.6%	0.20 [0.10, 0.40]
**Total (95% CI)**			**100.0%**	**0.47 [0.19, 1.20]**
Heterogeneity: Tau^2 = 0.66; Chi^2 = 15.23, df = 3 (P = 0.002); I^2 = 80%				
Test for overall effect: Z = 1.58 (P = 0.11)				

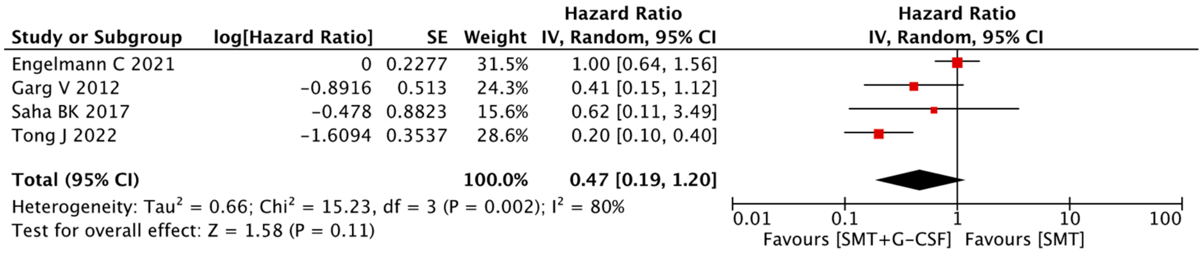

Engelmann C [19], Garg V [30], Saha BK [31], Tong J [32]

b) Overall survival, sensitivity analysis follow-up 60 days

Study or Subgroup	log[Hazard Ratio]	SE	Weight	Hazard Ratio IV, Random, 95% CI
**3.1.1 Overall Survival, main analysis**				
Duan XZ 2013	−0.6539	0.3737	19.6%	0.52 [0.25, 1.08]
Engelmann C 2021	0.0198	0.1921	32.7%	1.02 [0.70, 1.49]
Garg V 2012	−0.821	0.4389	16.3%	0.44 [0.19, 1.04]
Saha BK 2017	−0.6931	0.8212	6.4%	0.50 [0.10, 2.50]
Tong J 2022	−0.7985	0.2904	25.0%	0.45 [0.25, 0.80]
**Subtotal (95% CI)**			**100.0%**	**0.61 [0.39, 0.94]**
Heterogeneity: $Tau^2 = 0.12$; $Chi^2 = 8.08$, df = 4 (P = 0.09); $I^2 = 50\%$				
Test for overall effect: Z = 2.22 (P = 0.03)				
**Total (95% CI)**			**100.0%**	**0.61 [0.39, 0.94]**
Heterogeneity: $Tau^2 = 0.12$; $Chi^2 = 8.08$, df = 4 (P = 0.09); $I^2 = 50\%$				
Test for overall effect: Z = 2.22 (P = 0.03)				
Test for subgroup differences: Not applicable				

Hazard Ratio
IV, Random, 95% CI

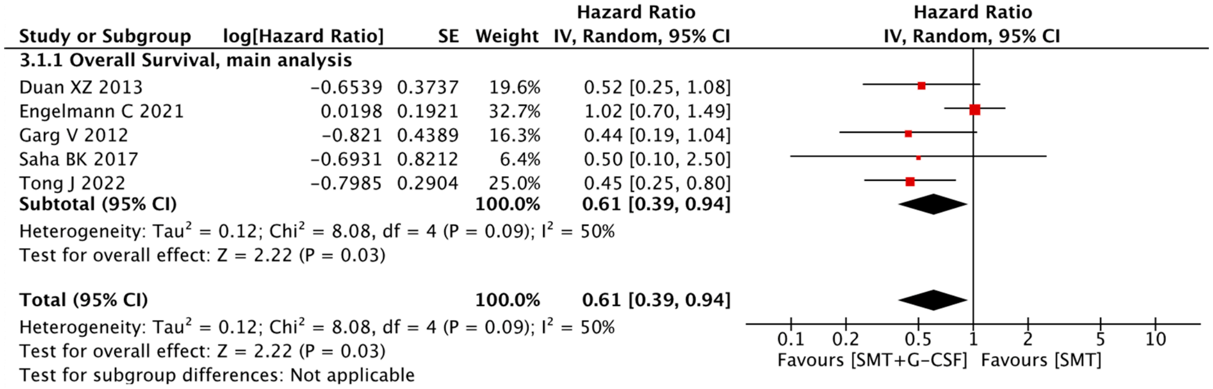

Duan XZ [29], Engelmann C [19], Garg V [30], Saha BK [31], Tong J [32]

c) Overall survival, sensitivity analysis follow-up 90 days

Study or Subgroup	log[Hazard Ratio]	SE	Weight	Hazard Ratio IV, Random, 95% CI
**3.1.1 Overall Survival, main analysis**				
Duan XZ 2013	−0.6733	0.3737	23.2%	0.51 [0.25, 1.06]
Engelmann C 2021	0.0488	0.1996	38.8%	1.05 [0.71, 1.55]
Garg V 2012	−0.821	0.4389	0.0%	0.44 [0.19, 1.04]
Saha BK 2017	−0.9163	0.7674	8.2%	0.40 [0.09, 1.80]
Tong J 2022	−0.6349	0.2904	29.8%	0.53 [0.30, 0.94]
**Subtotal (95% CI)**			**100.0%**	**0.67 [0.42, 1.07]**
Heterogeneity: $Tau^2 = 0.11$; $Chi^2 = 6.00$, df = 3 (P = 0.11); $I^2 = 50\%$				
Test for overall effect: Z = 1.68 (P = 0.09)				
**Total (95% CI)**			**100.0%**	**0.67 [0.42, 1.07]**
Heterogeneity: $Tau^2 = 0.11$; $Chi^2 = 6.00$, df = 3 (P = 0.11); $I^2 = 50\%$				
Test for overall effect: Z = 1.68 (P = 0.09)				
Test for subgroup differences: Not applicable				

Hazard Ratio
IV, Random, 95% CI

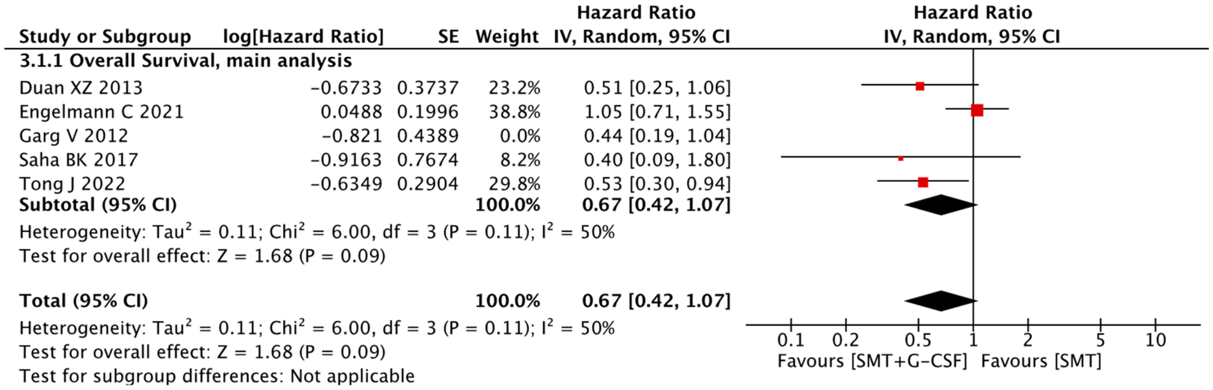

Duan XZ [29], Engelmann C [19], Garg V [30], Saha BK [31], Tong J [32]

Suppl.Figure 4. Overall survival, sensitivity analysis with competing risk analysis data

Study or Subgroup	log[Hazard Ratio]	SE	Weight	Hazard Ratio IV, Random, 95% CI
**3.1.1 Overall Survival, main analysis**				
Duan XZ 2013	−0.6733	0.34	21.2%	0.51 [0.26, 0.99]
Engelmann C 2021	0.1044	0.1866	30.9%	1.11 [0.77, 1.60]
Garg V 2012	−0.82	0.44	16.3%	0.44 [0.19, 1.04]
Saha BK 2017	−0.92	0.77	7.4%	0.40 [0.09, 1.80]
Tong J 2022	−0.6349	0.2904	24.1%	0.53 [0.30, 0.94]
**Subtotal (95% CI)**			**100.0%**	**0.63 [0.40, 0.99]**
Heterogeneity: $Tau^2 = 0.14$; $Chi^2 = 9.27$, df = 4 (P = 0.05); $I^2 = 57\%$				
Test for overall effect: Z = 1.99 (P = 0.05)				
**Total (95% CI)**			**100.0%**	**0.63 [0.40, 0.99]**
Heterogeneity: $Tau^2 = 0.14$; $Chi^2 = 9.27$, df = 4 (P = 0.05); $I^2 = 57\%$				
Test for overall effect: Z = 1.99 (P = 0.05)				
Test for subgroup differences: Not applicable				

Hazard Ratio
IV, Random, 95% CI

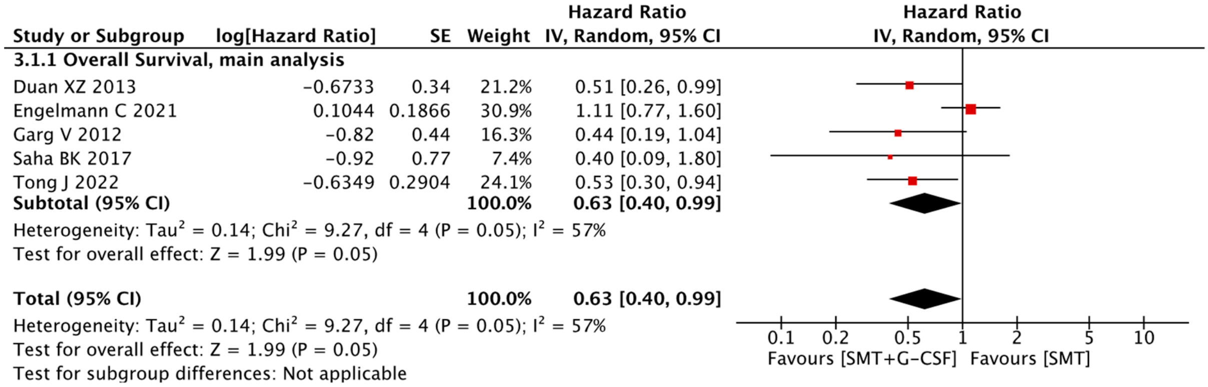

Duan XZ [29], Engelmann C [19], Garg V [30], Saha BK [31], Tong J [32]

Suppl.Figure 5. MELD score, sensitivity analysis follow-up 30 days

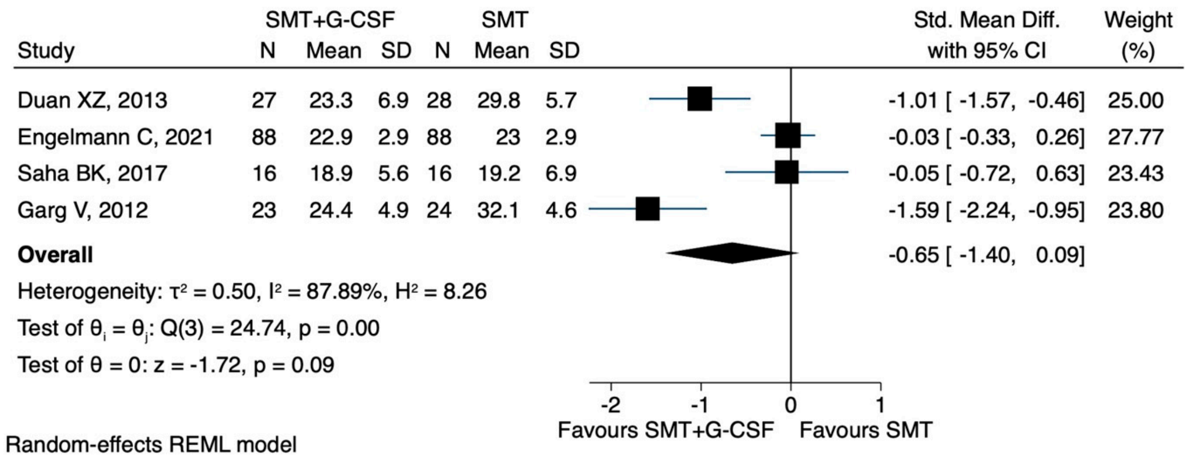

Duan XZ [29], Engelmann C [19], Garg V [30], Saha BK [31]

Suppl.Figure 6. MELD score, sensitivity analysis risk of bias

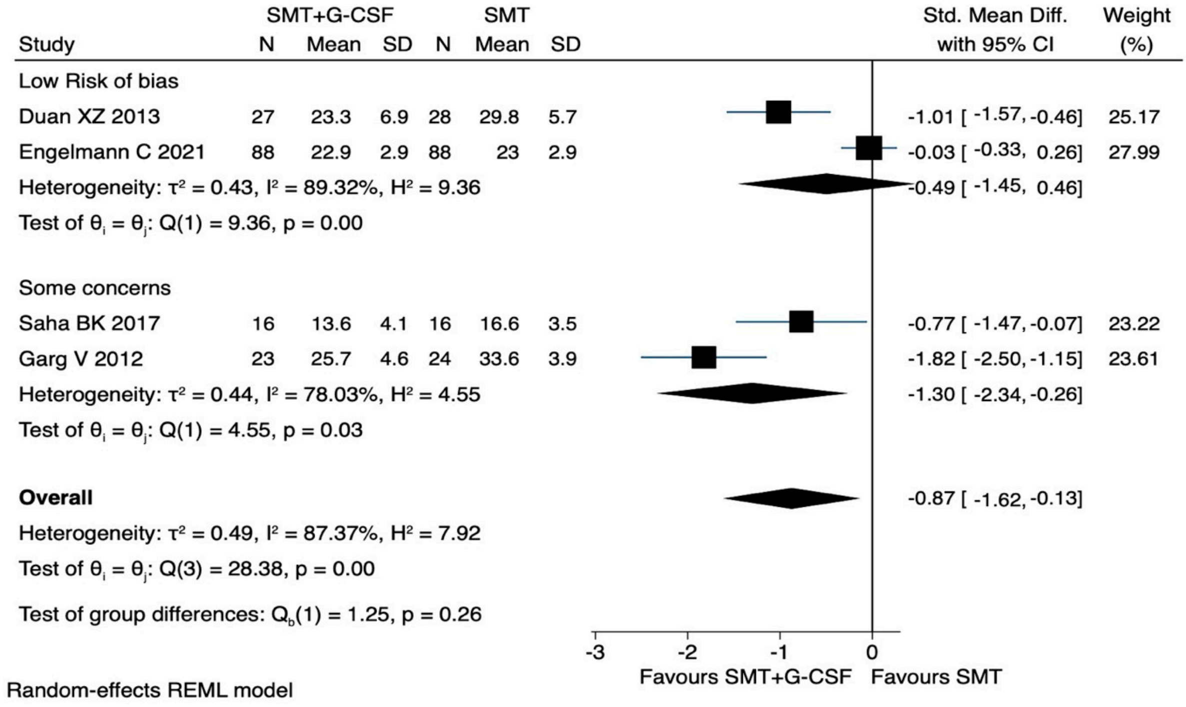

Duan XZ [29], Engelmann C [19], Garg V [30], Saha BK [31]

Suppl.Figure 7. MELD score, sensitivity analysis ACLF criteria

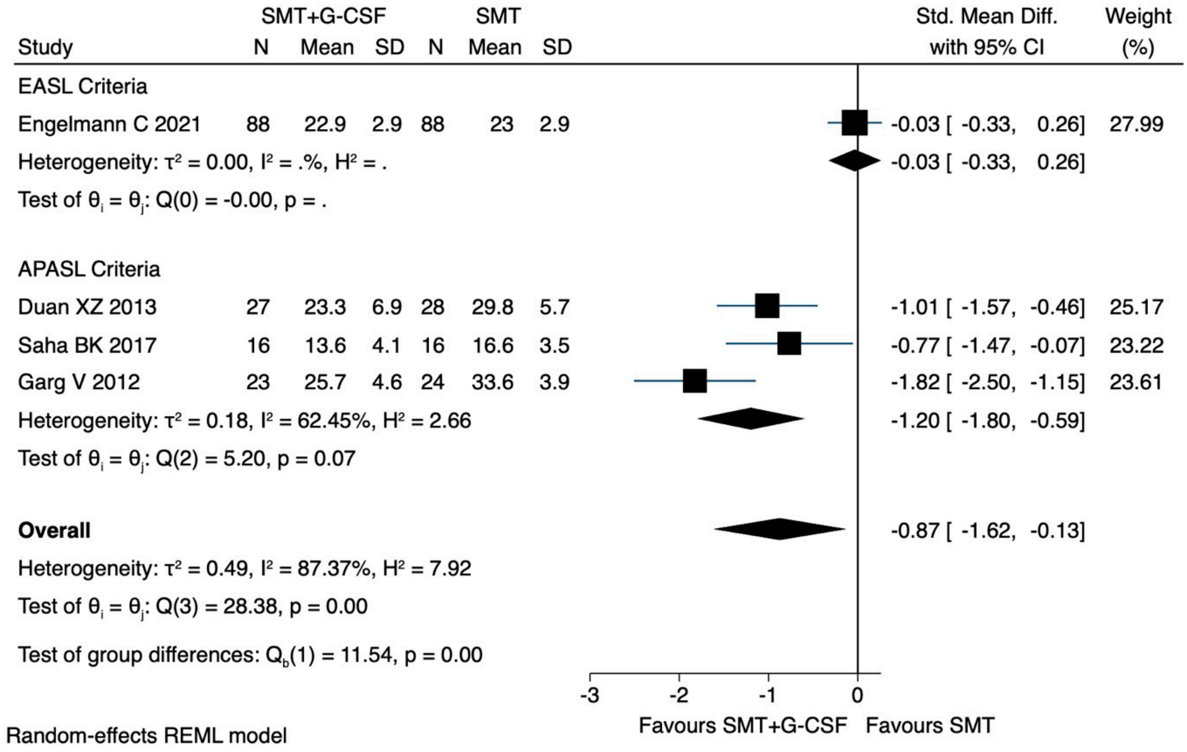

Duan XZ [29], Engelmann C [19], Garg V [30], Saha BK [31]

Suppl.Figure 8. MELD score, post-hoc analysis available case

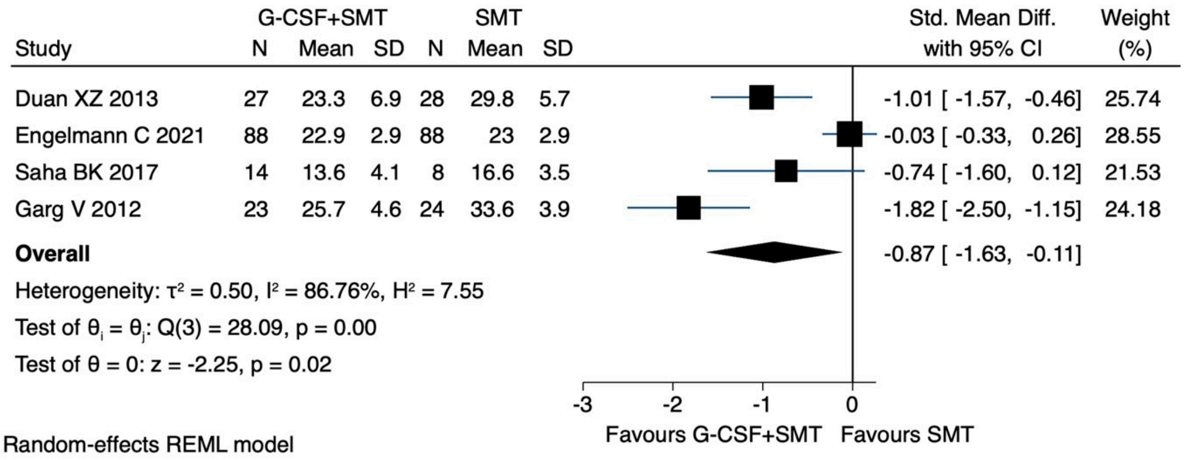

Duan XZ [29], Engelmann C [19], Garg V [30], Saha BK [31]

Suppl.Figure 9. Child-Pugh score, sensitivity analysis statistical values

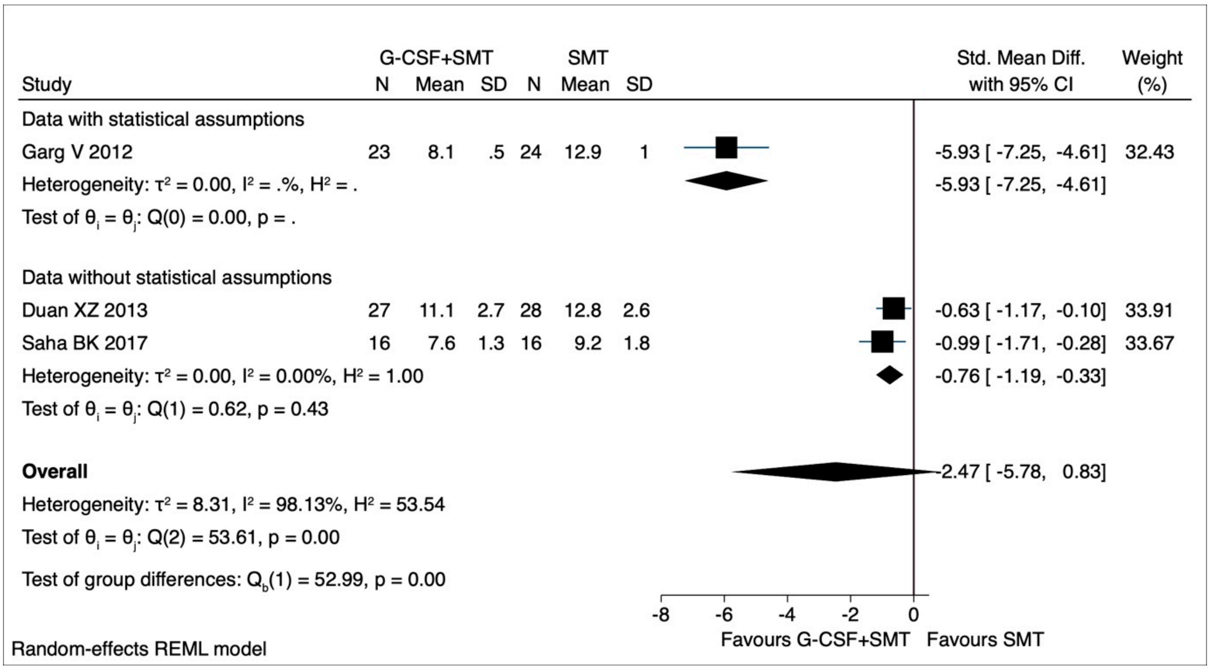

Duan XZ [29], Garg V [30], Saha BK [31]

Suppl.Figure 10. Child-Pugh score, sensitivity analysis 30 days follow-up

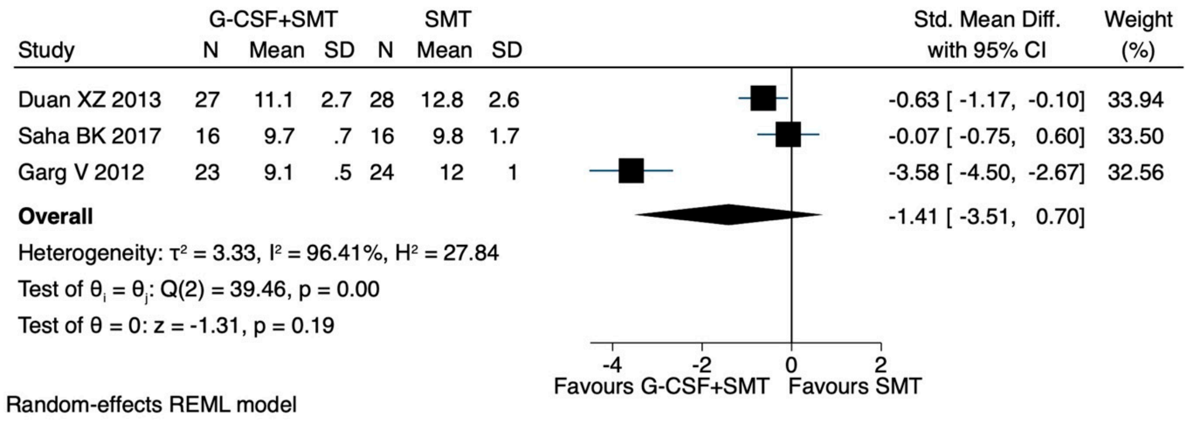

Duan XZ [29], Garg V [30], Saha BK [31]

Suppl.Figure 11. Child-Pugh score, post-hoc analysis available case

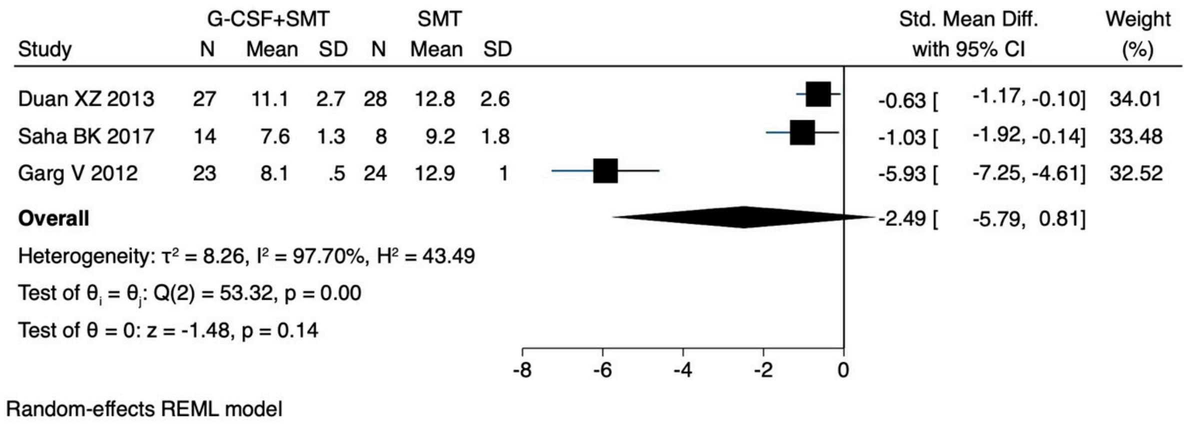

Duan XZ [29], Garg V [30], Saha BK [31]

Suppl.Figure 12. Mortality, sensitivity analysis EASL criteria

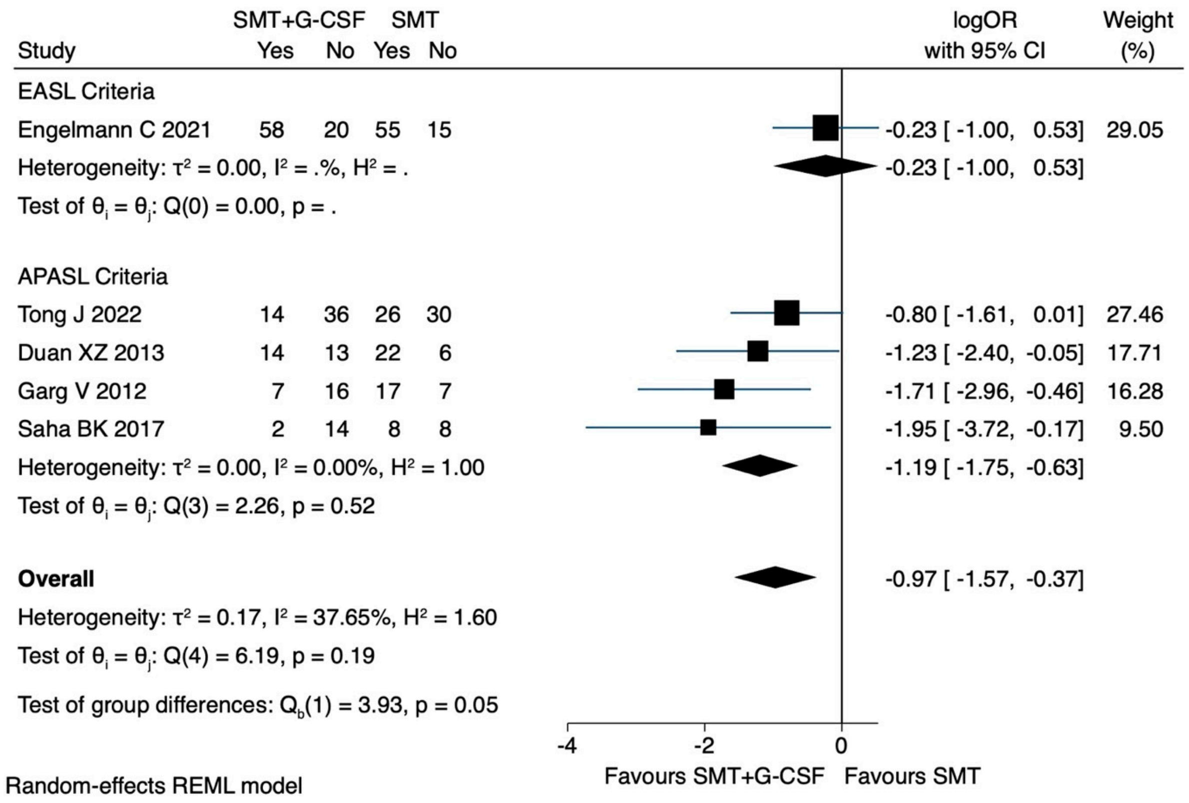

Duan XZ [29], Engelmann C [19], Garg V [30], Saha BK [31], Tong J [32]

Suppl.Figure 13. Mortality, sensitivity analysis risk of bias

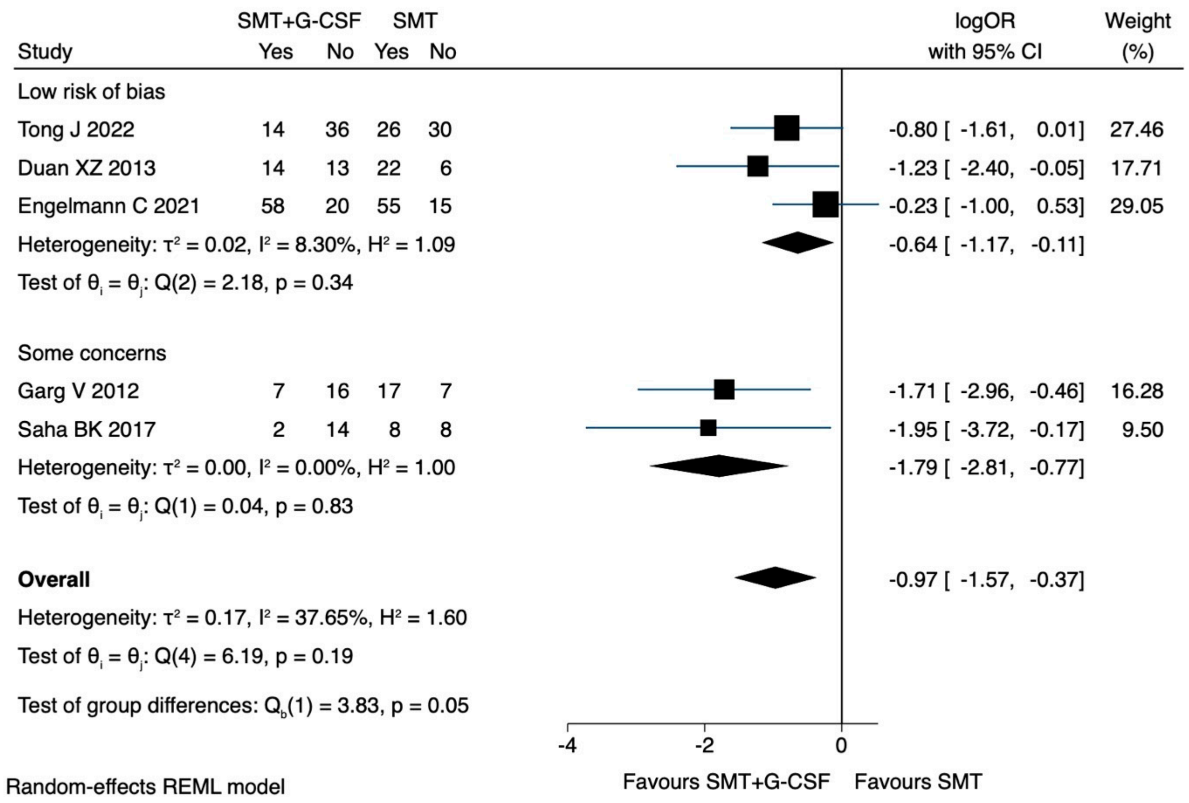

Duan XZ [29], Engelmann C [19], Garg V [30], Saha BK [31], Tong J [32]

Suppl.Figure 14. Cirrhotic complications, sensitivity analysis risk of bias

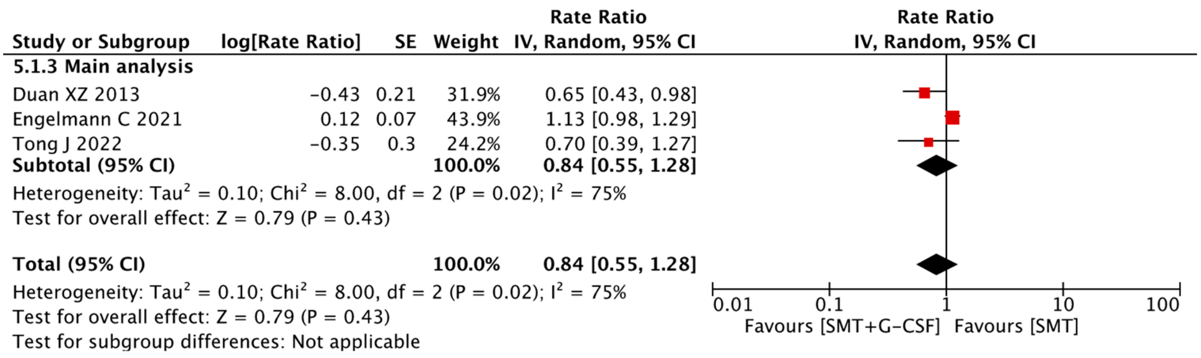

Duan XZ [29], Engelmann C [19], Tong J [32]

Suppl.Figure 15. Cirrhotic complications, sensitivity analysis EASL criteria

Study or Subgroup	log[Rate Ratio]	SE	Weight	Rate Ratio IV, Random, 95% CI
**5.1.3 Main analysis**				
Duan XZ 2013	−0.43	0.21	31.1%	0.65 [0.43, 0.98]
Garg V 2012	−1.77	0.33	27.2%	0.17 [0.09, 0.33]
Saha BK 2017	−1.5	0.79	13.6%	0.22 [0.05, 1.05]
Tong J 2022	−0.35	0.3	28.2%	0.70 [0.39, 1.27]
**Subtotal (95% CI)**			**100.0%**	**0.40 [0.19, 0.83]**
Heterogeneity: $Tau^2 = 0.41$; $Chi^2 = 14.53$, df = 3 (P = 0.002); $I^2 = 79\%$				
Test for overall effect: Z = 2.45 (P = 0.01)				
**Total (95% CI)**			**100.0%**	**0.40 [0.19, 0.83]**
Heterogeneity: $Tau^2 = 0.41$; $Chi^2 = 14.53$, df = 3 (P = 0.002); $I^2 = 79\%$				
Test for overall effect: Z = 2.45 (P = 0.01)				
Test for subgroup differences: Not applicable				

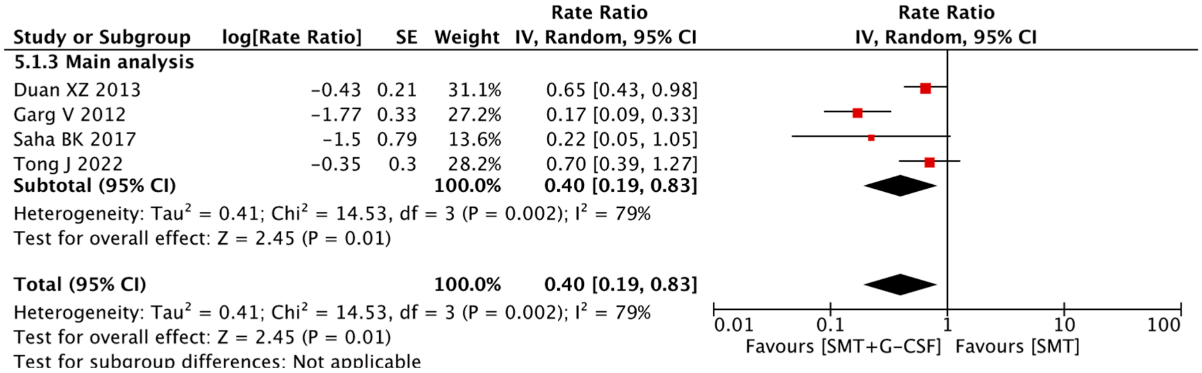

Duan XZ [29], Garg V [30], Saha BK [31], Tong J [32]

Supplementary Table 1: Risk of bias assessment for primary outcome overall survival with Cohrane RoB tool 2.0

First Author, Year, Country	Randomization process	Deviations from intended intervention s	Missing outcome data	Measure ment of the outcome	Selection of the reported result	Overall Bias
Saha BK, 2017, India	Some concerns	Low	Low	Low	Low	Some concerns
Engelmann C, 2021, Germany	Low	Low	Low	Low	Low	Low
Duan XZ, 2013,China	Low	Low	Low	Low	Low	Low
Garg V, 2021, India	Some concerns	Low	Low	Low	Low	Some concerns
Tong J, 2022, China	Low	Low	Low	Low	Low	Low

Duan XZ [29], Engelmann C [19], Garg V [30], Saha BK [31], Tong J [32]

Saha BK [31], 2017, India :

Randomization: The grouping was done as per the visit of the patients to the hospital
Deviations from intended interventions: Low
Missing outcome data: sufficient information has been provided
Measurement of the outcome: Low risk
Selection of the reported result: Low risk

Engelmann C[19], 2021, Germany

Randomization: Web-based allocation sequence generation
Deviations from intended interventions: Low
Missing outcome data: sufficient information has been provided

Measurement of the outcome: Low risk
Selection of the reported result: Low risk

Duan XZ[29], 2013, India

Randomization: double blind, placebo-controlled
Deviations from intended interventions: Low risk
Missing outcome data: ITT analysis
Measurement of the outcome: Low risk
Selection of the reported result: Low risk

Garg V[30], 2021, India

Randomization: Some concerns
Deviations from intended interventions: Low risk
Missing outcome data: Reasons for missing outcome data unlikely to be related to true outcome
Measurement of the outcome: Low risk
Selection of the reported result: Low risk

Tong J[32], 2022, China

Randomization: randomized allocation sequence generation
Deviations from intended interventions: Low
Missing outcome data: sufficient information has been provided
Measurement of the outcome: Low risk
Selection of the reported result: Low risk

Suppl.Table 2, Main differences between APASL and EASL criteria

**Parameters**	**APASL definition**	**AASLD/EASL working party definition**
Duration between acute insult and ACLF	Four weeks	Not defined
Underlying chronic liver disease	Non-cirrhotic chronic liver disease and only compensated cirrhosis	Only cirrhosis, non-cirrhotic chronic liver disease excluded
Acute precipitating event	Alcohol, drugs, hepatotropic viruses, surgery, trauma	Alcohol, drugs, hepatotropic viruses, surgery, trauma, infection/sepsis, variceal bleed
Organ Failure	Hepatic failure	Extra-hepatic organ failure
